# It Takes a Village: Partnerships in Primary School Relationships and Sexuality Education in Aotearoa

**DOI:** 10.1007/s40841-022-00260-5

**Published:** 2022-07-28

**Authors:** Rachael Dixon, Tracy Clelland, Megan Blair

**Affiliations:** Faculty of Health, University of Canterbury, Christchurch, New Zealand

**Keywords:** Relationships & sexuality education, Health education, Home-school partnerships, Appreciative Inquiry

## Abstract

The implementation of relationships and sexuality education as part of Health and Physical Education in *The New Zealand Curriculum* (Ministry of Education, Ministry of Education. (2007). The New Zealand Curriculum. Learning Media Limited.) involves a range of people sharing their perspectives in order to shape the subject on paper and in practice. This paper presents the findings of a qualitative collective case study in three primary schools in Aotearoa. Experimenting with Appreciative Inquiry, we found that connections and conversations between a wide variety of people and organisations have a critical role to play in relation to planning and teaching relationships and sexuality education in schools: (i) Schools and teachers working in partnership with colleagues within and across schools, (ii) connections with whānau and relationships with learners, and (iii) access to wider supports and services. Our findings suggest that having conversations and establishing and maintaining productive partnerships between a variety of people are critical if relationships and sexuality education is to live up to its potential and meet learners’ needs.

## Introduction

Human connection is at the heart of sexuality education and the implementation and adaptation of the school curriculum over time inevitably involves a range of people sharing their perspectives and working together in order to shape the subject on paper and in practice. Sexuality education is named as one of seven key areas of learning within the Health and Physical Education learning area (HPE) in *The New Zealand Curriculum* (NZC) (Ministry of Education, 2007) as well as in the curriculum document which preceded the NZC, *Health and Physical Education in the New Zealand Curriculum* (Ministry of Education, 1999). Learning is mandated from year 1 to year 10 of the NZC in all learning areas, which means learning experiences in sexuality education should exist from the beginning of a child’s schooling journey.

In 2002, the Ministry of Education published guidance for school leaders, teachers, and school boards to support the effective implementation of sexuality education. A 2013 report of the Health Select Committee into improving child health outcomes (Hutchison, 2013) unequivocally made a case for (i) strengthening sexuality education in schools and (ii) monitoring of schools’ programmes by the Education Review Office (ERO). This report was followed in 2015 by the Ministry of Education’s update to the 2002 guidelines as well as a national evaluation into sexuality education in schools by ERO which found significant gaps in sexuality education in some schools (ERO, 2018).

Amidst a backdrop of cultural and social changes including the proliferation of social media and young people’s use of digital devices, changing family structures, and changing social and gender norms, the 2015 sexuality education guide was re-developed and published in 2020 as *Relationships and Sexuality Education: a guide for teachers, leaders and boards of trustees* (from here, the RSE guide) (Ministry of Education, 2020a). Two significant aspects of this re-development are the re-naming of the area of learning as relationships and sexuality education (RSE) and the separation of the guide into two documents: one for years 1–8 (primary) and one for years 9–13 (secondary) (Fitzpatrick et al., 2021). Both of these movements extend opportunities for primary schools to incorporate RSE into learning programmes from year 1 of the curriculum, in partnership with their school communities.

As suggested by the 2018 ERO evaluation (ERO, 2018), implementation of RSE is inconsistent across primary schools in the country. Boyd and Hipkins (2015) state “unlike other learning areas, HPE has goals and ways of working that overlap with the wider mission and ways of working of schools” (p. 10). This is evidenced in RSE through the involvement of the health sector and interest groups in resourcing and teaching in health education classrooms. It is well documented that a number of externally-provided programmes and facilitators are entrenched in HPE in Aotearoa^1^ schools, particularly at the primary school levels (Petrie et al., 2014; Powell, 2015). However it is unclear as to the extent of this occurring in RSE specifically, and how external providers partner with schools to support RSE.

Health education (and RSE within) is the only subject in the curriculum for which schools must consult their community as outlined in the Education and Training Act 2020 (New Zealand Government, 2020). This consultation must take place every two years and involves consultation on how health education as a subject is to be delivered in a school. While anecdotal evidence suggests that the majority of school community members (including whānau^2^) support schools’ plans for health education, research evidence is needed as to teachers’ and school leaders’ understanding of the consultation requirements and means by which they consult with their communities.

The purpose of this paper is to explore partnerships in primary school RSE in Aotearoa. Presenting findings from a qualitative study using case studies of three primary schools, we negotiate territory relating to who should be involved and have a say in RSE, how discussions between people might be facilitated and partnerships formed, and what possibilities might exist for thinking differently about how we work with people in RSE. We do this with the hope of igniting thought about how we can work as a village to plan for, and enact, RSE that meets the needs of learners in primary schools in Aotearoa.

## Literature

Pertinent literature relating to partnerships between members of the village involved in RSE in primary schools in Aotearoa traverses the following areas: Home-school partnerships, the role of external providers and support services in RSE, and community consultation in relation to health education in the Education and Training Act 2020 (New Zealand Government, 2020). We limited our search for literature to Australian and Aotearoa publications, given the similar curriculum context for RSE in the two countries.

RSE in primary schools is an under-explored area in Aotearoa research, which led us to look more broadly towards Ministry of Education policy documents, home-school partnerships literature, and ERO’s evaluative work, which is relevant across learning areas in the NZC. Newly developed National Education and Learning Priorities in Aotearoa (Ministry of Education, 2020b) include objective one: Learners with their whānau are at the centre of education. Priority area two for this objective requires schools to partner with whānau and communities to provide responsive education. Providing a framework for this in the context of RSE, the RSE guide (Ministry of Education, 2020a) refers to a whole-school approach, which draws upon the health promoting schools model to delineate three areas of school life: Ethos and environment, curriculum teaching and learning, and community connections (Ministry of Education, 2020a). It is the latter dimension of school life that considers the importance of connections and partnerships with whānau, education and health agencies, and community groups.

The existence of productive and positive partnerships between schools and whānau make a positive difference to the engagement and achievement of learners (Bull et al., 2008; Highfield & Webber, 2021; Mutch & Collins, 2012). Participation by whānau in areas of school life has, over time, involved parents through decision-making (for example as elected members of a school’s board); participation and collaboration in school events; and information-sharing around school policies and practices, and teaching and learning (Mutch & Collins, 2012). Home-school partnerships have been found to be more effective when relationships are collaborative and mutually respectful, responsive to community needs, embedded within the school’s culture, focused upon learning, and based upon timely two-way communication (Bull et al., 2008; Mutch & Collins, 2012). Moreover, partnerships to support student engagement and achievement also involve wider community members, including local kaumatua (respected elder in a Māori community) and iwi (tribe) through local curriculum design and place-based learning (Highfield & Webber, 2021). Applying home-school partnerships to RSE specifically, ERO (2018) discussed the importance of connections to community, including the role of face-to-face hui (meetings) with whānau and communities, the need for information to be provided to whānau about RSE programmes, and opportunities for whānau to be involved in the two-yearly consultation for health education. In the Australian context, Walker et al. (2021) and Robinson et al. (2017) stress the importance of collaboration between schools and parents/families in order to plan for, and teach, a responsive RSE in primary schools. The Australian research asserts the need to partner with parents to assuage any fears over clashes in values between the school and the parent community, and to ascertain parental wishes for RSE (Robinson et al., 2017; Walker et al., 2021).

The role of external providers and support services in health education and RSE is a perennial issue in the literature across Aotearoa and Australia; including issues relating to the role and purpose of outside agencies (Johnson et al., 2014; Leahy et al., 2016; Petrie et al., 2014; Powell, 2015; Ministry of Education, 2020a; Walker et al., 2021) and how to create partnerships that enhance RSE learning for students in primary schools (ERO, 2018; Ministry of Education, 2020a; Tūturu, 2020). ERO (2018) asserts the importance of connection to external groups such as health services, the Police and non-governmental organisations working in the area of RSE (ERO, 2018) in ways that support teachers to plan for and teach RSE, rather than solely take on the role of the classroom teacher in RSE. The Ministry of Education (2020a) asserts that it is not best practice to hand over responsibility for RSE to external providers, but instead, for schools to use external providers to support teachers with specialist knowledge and expertise. In this way, teachers are involved in planning for, and teaching, RSE in ways that embed RSE across a period of time. To illustrate this approach, Johnson et al. (2014) researched primary school teachers’ confidence to teach RSE when supported with a 10-lesson unit of learning created by an RSE external provider. Using the unit led teachers to work in partnership with their colleagues to modify learning to meet their students’ needs, and ultimately feel more confident teaching RSE. The authors concluded that establishing partnerships with external providers, including providing materials for teaching RSE, can enhance RSE in primary schools. In Aotearoa, this connects to the work of Family Planning, a non-governmental organisation. Rather than taking on the teaching of students themselves, Family Planning provide support for teachers to teach RSE through their extensive teaching and learning resources, professional learning and development workshops with school staff, and guidance on strategies for effective teaching, learning, and assessment in RSE (Family Planning, n.d).

The Education and Training Act 2020 requirement to conduct community consultation around the delivery of health education (and RSE within) every two years (New Zealand Government, 2020) connects to home-school partnerships in terms of two-way communication and consultation. The Act offers wider community members and learners the opportunity to provide voice that helps shape the delivery of health education in a school (Ministry of Education, 2020a; Tūturu, 2020). The requirement for community consultation was written into the previous Education Act 1989, therefore the requirement to formally consult is not new. There is a lack of research, however, about schools’ mechanisms for, and experiences of, the health education consultation. ERO (2018) report that schools with effective practice in RSE maintained good connections with their community, including hui and face-to-face consultation, which resulted in RSE programmes that reflected the values of their communities. Guidance developed by the Ministry of Education (2020a) and Tūturu (2020) explicates for schools the legal requirements for consultation, and offers ideas and resources for consulting. Both documents refer to the need to consult not only whānau, but teachers in the school, external providers and other community members, and—last but not least—learners.

As the review of literature above indicates, a range of research and guidance documents exist in the area of home-school partnerships, the role of external providers and support services in RSE, and community consultation in relation to health education in the Education and Training Act 2020 (New Zealand Government, 2020). However, a gap exists in relation to how all of this ‘plays out’ in practice in Aotearoa primary schools, particularly in relation to RSE.

## An Appreciative, Socially Constructed Approach to Inquiry

Our research is underpinned by an Appreciative Inquiry approach. Appreciative Inquiry is grounded in social constructionism, through shared sense-making (Clouder & King, 2015) as researchers and research participants learn from each other about what is valuable about practices and how this value can be built upon. In context of our inquiry, we acknowledge that RSE practices in primary schools are bound by cultural, social, political, and historical contexts unique to different schools. It is through conversations with participants that we, as researchers, can draw out strengths in RSE practice as well as spark conversations about possibilities for building on these strengths.

Appreciative Inquiry was developed to reveal often overlooked positive aspects of experience, generate theory about ‘what works’ in practice, and plan for a new reality. An Appreciative Inquiry agenda looks beyond what is broken to prioritise the positive and discover and generate stories of success (Enright et al., 2014) and appreciates people’s strengths rather than focusing on their shortcomings (Clouder & King, 2015). We view this as a more productive approach when working with RSE teachers and leaders: appreciating the strengths of their practice is more likely to facilitate rich conversations and support them to build upon these strengths moving forward.

The 4D model, from Cooperrider and Whitney (2000) is a common model used to frame methods and data collection questions (Sargent & Casey, 2021). The 4D model comprises the stages of discovery, dream, design, and destiny. Positioning our study as exploratory, we have focused upon the first two stages in the model – discovery (what’s going well in RSE?) and dream (what are your hopes for RSE?). By framing our research inquiry towards an appreciative agenda, we have the opportunity to hear and tell stories about what’s going well in RSE practice and how we can learn from this in order to capitalise upon the rich potential of RSE learning and whole school approaches that affirm children’s and young people’s identities and support the development of knowledge and skills for healthy relationships and sexual health. Moreover, as researchers we can assess the feasibility of working with the 4D model in its entirety for future research projects.

## Methods

The wider study from which this paper is drawn was a multi-phase, mixed methods inquiry, which for phase one involved a nationwide survey of primary principals and teachers. In this paper, we focus on findings from phase two of our study: Collective case study in three primary schools. The purpose of using collective case study design was to inquire in-depth, and with an appreciative lens, RSE practice in schools with primary-aged learners. As a collective case study, we are able to analyse data across social settings that share commonalities (Goddard, 2010). Below, we provide details of participants, data collection methods, approach to data analysis, and methodological issues.

Ethical approval for the research was gained from the University of Canterbury’s Human Research Ethics Committee.

### Participants

Case study schools were recruited through a question in our nationwide survey asking whether those completing the survey would be interested in participating in a group interview (hui) with the researchers. This resulted in six schools contacting us, and three case schools were finally chosen due to the schools’ and our availability to schedule and conduct the hui before the end of the 2021 school year.

School 1 is a state composite school (years 1–13) with under 700 students. Participating in the hui were the teacher in charge of health education and two teachers of years 7–8 learners. School 2 is a special character full primary school (years 1–8) with under 100 students. Participating in the hui were the principal and two teachers. School 3 is a state contributing primary school (years 1–6) with under 500 students. Participating in the hui were the deputy principal, the teacher with curriculum responsibility for health education, a teacher of years 1–2 learners, a teacher of years 3–4 learners, and a teacher of years 5–6 learners.

### Data Collection

Data collection for our case study phase of the research consisted of in-depth semi-structured interviewing and a workshop approach that involved participants working with extracts from the RSE guide (Ministry of Education, 2020a) and pre-prepared prompts to provoke dialogue between participants. The selected extracts from the RSE guide (Ministry of Education, 2020a) were (in the order in which we used these in the hui): A whole-school approach to RSE, key learning charts across curriculum levels 1–4, and RSE for diverse ākonga.^3^ The three hui were between 90 min and 2 h long. The hui were audio recorded, with written transcripts created for analysis. Supporting the interview transcripts were notes from participants’ involvement in the workshop activities.

### Data Analysis

Reflexive thematic analysis was approached both inductively and deductively for our case study data. Inductive analysis enabled us to code the data in a way that was open to the possibilities presented in the transcripts. Deductive analysis enabled us to code the data in relation to our Appreciative Inquiry theoretical framework, as well as our pre-existing understanding of RSE, which included common challenges, tensions and possibilities documented in RSE literature with which we were familiar.

Our thematic analysis for the case studies data was undertaken both individually and collectively. We worked together to familiarise ourselves with the data, before independently coding and generating initial themes. We then came back together to revise and refine themes, before defining and naming themes. Working in this way allowed us not to achieve consensus, but to add to each person’s analytical thoughts to create a richer analysis overall (Braun & Clarke, 2021).

## Analysis of Findings: It Takes a Village

### Introduction

In our analysis of data, we found a range of connections and conversations between a wide variety of people and organisations that had a critical role to play in relation to RSE both inside and outside the classroom. Planning and teaching RSE involved:

(i) Schools and teachers working in partnership with colleagues within and across schools
(ii) Connections with whānau and relationships with learners
(iii) Access to wider supports and services.

In combination, these partnerships, although presenting challenges at times, offered our participants the guidance and reassurance that they needed to plan and teach RSE that was responsive to learners’ needs.

### Collaboration With Colleagues Within and Across Schools

Participants in our case study schools recognised the importance of collegiality and having opportunities to learn from and with more experienced colleagues. This was particularly the case for teachers of younger learners, who in the past had not taught RSE at the lower levels of the curriculum, as Hannah from School 2 exemplifies:

I’ve always taught juniors, mostly… It’s been oh its puberty, it’s the older part of the school… so I do feel a wee bit out of my depth a wee bit because I haven’t…

Strategies to support each other as teaching staff were discussed, for example: “Time to sit together and discuss and to look really closely at the lessons and have those important conversations with each other, raise any concerns” (Christine, School 2).

Collegiality was also discussed in relation to teachers’ differing comfort levels with RSE:

I think we’ve been mindful of people’s religious beliefs. We have said if you feel uncomfortable or if you need somebody to support you… we ask them what they need, we don’t just demand that they teach something that they’re uncomfortable with… So, we do seek ways to find solutions to any uncomfortableness for anybody. (Alison, School 3).

Here, Alison recognises that teachers bring different levels of experience and comfort to their role as a teacher of RSE, and that a supportive school environment is needed to enable teachers to provide quality RSE (Ministry of Education, 2020a; Walker et al., 2021). This connects to the constructivist principle of appreciative inquiry in that teachers can work in dialogue with colleagues in order to develop confidence and strengthen practice.

The issue of support (or lack thereof) from senior leadership and governance (the school board) also came to the fore. Different experiences were discussed across the schools when undertaking the workshopping component of the hui (prompt one: a whole-school approach). The perception existed that senior leadership did not prioritise RSE or health education: “It hasn’t always been the top priority for our school management, I guess” (Joanne, School 1). In School 3, however, connections between governance, policies, and teacher practices were articulated:

So, your board, they know everything that’s gone through your policies and procedures, and that’s spoken about at every board meeting… Staff know about neglect or abuse—they know about those policies—we go over those (Alison).

Support from school leadership for RSE and health education is widely discussed in the literature as a key factor influencing teaching and learning (ERO, 2018; Ministry of Education, 2020a; Walker et al., 2021). Commentary from our participants resonates with this, indicating that teacher practice in RSE is constrained or enabled by the value and priority afforded to the subject by leaders in a school.

Connections across schools were also mentioned in the case study schools as being valuable when planning for learning in RSE. For School 2, this came about through connecting with other special character schools in the local area including the secondary school:

(We) brainstormed everything… transition(s) into their school, because they’re our main feeder school (secondary school) in (region) for the Catholic system (Christine).

Here, Christine discusses how the secondary school health education teacher enabled the feeder schools an opportunity to get together, share knowledge and discuss issues pertinent to Catholic schools in the local area. Kāhui Ako (communities of learning) provide a government-funded mechanism whereby groups of schools work together to help students reach their full potential across their learning pathway from early childhood to secondary school education (Highfield & Webber, 2021; Ministry of Education, 2021). Two of the three schools participating in our research were part of Kāhui Ako, however the potential of these partnerships for connecting schools in curriculum areas such as health education could be further developed, as Rebecca from School 1 noted:

There’s so many different things that people are doing and they don’t ever really get shared

with everyone going in that same direction… Nobody else knows about it and it just doesn’t become this whole school approach.

This comment points to the fact that teachers are perennially juggling many tasks, and whole-school and wider school initiatives can become lost amid everything else that needs to be done. Nevertheless, the Kāhui Ako model presents a valuable opportunity for schools in similar geographical locations or with shared special characters to work in partnership in areas such as RSE to enable robust learning pathways and to share knowledge and expertise, including around connecting to whānau in the community.

### Connections With Whānau

Regular and transparent communication with whānau around matters to do with RSE were discussed extensively across the interviews with the case study schools. Participant commentary traversed the territory from informing parents and whānau, to seeking their input on RSE learning, and the challenges that arose periodically with whānau views and understandings around RSE.

When informing parents about RSE in their school, participants were often ready to receive negative feedback and withdrawals from class, but this more often than not did not transpire. Sexuality education is the one part of *The New Zealand Curriculum* (Ministry of Education, 2007) where parents can request for their child to be withdrawn (New Zealand Government, 2020). Joanne (School (1) noted that “we’ve had a couple of kids across the 7/8 s withdrawn but very few.”

Participants across the three hui discussed how they front-footed communications to whānau about up-coming RSE learning. This helped reassure teachers that whānau supported what was being taught and enhanced the opportunity to initiate conversations at home with their children to reinforce learning at school. This is exemplified in the following exchange from the participants from School 3:

Some of it is what we perceive as going to be an issue for parents and it’s not. They are so pro teaching body parts and everything, they just want to know what we’re teaching. (Alison).

We’ve had no negative feedback have we… We communicate regularly and we kind of give the parents a heads up about what’s sort of coming next and there’s definitely been conversations happening at home with children which has been really positive—so prior to the lessons being delivered (Liz).

The importance of open communication with whānau is consistently recognised across RSE research (ERO, 2018; Robinson et al., 2017; Walker et al., 2021) and guidance (Ministry of Education, 2020a) and home-school partnership literature more broadly (Bull et al., 2008; Highfield & Webber, 2021; Mutch & Collins, 2012).

While open communication with whānau is vital to responsive RSE, schools discussed the importance of being inclusive with diverse communities. Rebecca (School 1) commented that the school has “such diverse kids and therefore the whānau community should represent that as well and their voice is important.” Communication with whānau was seen as valuable for many reasons, including to dispel assumptions or misunderstandings about RSE: “they still believe that sexuality education is just about sex, so it’s educating the parents that that’s not the case” (Rebecca, School 1). The participants in School 3 discussed seeking input from families of Muslim students at the school:

We have quite a large Muslim community at (school) too, so we did make sure that there was a place for them to ask questions because we did know that they may be one of the communities that might … (Miriama).

They might just have had a few more concerns about the content that we were sharing. But they didn’t, they were very, very happy, predominantly, with what we were doing anyway, but they had lots of questions (Alison).

The extracts above speak to the sometimes challenging aspects of seeking input from whānau, such as navigating different cultural attitudes and values, and dispelling misunderstandings of RSE. The extract above supports the notion that whānau from diverse cultural backgrounds welcome opportunities to hear and ask questions about the RSE programme.

Issues relating specifically to the mandated community consultation were discussed across the case study hui. Rebecca from School 1 indicated that her school had, until recently, not been up-to-date with the consultation requirements:

I kind of had to drive this from my position on the board, but also my passion and advocacy for health education and so the unfortunate thing is (the consultation) hasn’t been done until now… And then I got on the board and then kind of, well it happened, and I think (principal) helped as well, advocate for that.

Here, issues relating to governance and leadership can either be a barrier or enabler to community consultation. According to ERO (2018) leaders have a crucial role in ensuring effective stewardship in a school. Rebecca’s comment above attested that having a principal and a teacher on (the) board makes a difference in relation to the mandated consultation.

Each school offered information about the ways in which they had recently consulted, including complications caused by COVID-19 restrictions in the current and past year. For School 1, face-to-face consultation was highly valued:

We feed off the face-to-face communication as well a lot of the time. You know seeing the people respond and just having the communication and conversation just evolve naturally (Emma).

I like the idea of an information evening and then a bit of discussion afterwards and finding out what people think once they’ve been informed what’s actually in the programme rather than assuming they know what’s in it (Joanne).

School 3’s plans for consultation in the current year had been skittled by COVID-19 restrictions:

This year we were going to run (a meeting) for all parents across the school, which we didn’t end up being able to run just because they weren’t allowed on site (Miriama).

Participants’ comments above indicate that they understood the need for community consultation around health education (including RSE), and are cognisant of the need for face-to-face, on-going consultation activities with a variety of community members in order to best enact the consultation (Ministry of Education, 2020a; Tūturu, 2020).

The teachers were, however, grappling with the question of how much information they should provide as part of the community consultation, as well as how to explain to whānau the purpose of the community consultation. The question of how much information is too much information was discussed by Christine from School 2:

We don’t want them to feel like by getting all the information they can pick and choose what they want taught to their children. And it’s not normally about hiding something from parents, it’s about, either they can withdraw their children if they want, but sometimes if you give everything that’s when they start going no, we don’t want it taught… So that was the sort of way we were debating it—what’s going to be helpful to parents and what’s actually going to cause anxiety that they didn’t need to have in the first place.

While Alison from School 3 discussed how whānau were surprised about being asked for input into the RSE programme:

The parents came and said we were surprised you talked to us about it because you don’t talk to us about reading or maths—you don’t consult around how we deliver that programme and yet you’re asking us about this.

According to Robinson et al. (2017), schools need to provide parents with more information about what is taught in RSE, for example an outline of the curriculum across levels of schooling. The mandated community consultation in Aotearoa thus provides schools and teachers a useful mechanism for opening conversations with whānau about RSE.

### Relationships With Learners

Participants in the case study schools discussed the importance of the need for well-established relationships with learners before RSE was to take place. The value of having well-established relationships with learners was explored across the hui. The way in which classes were structured at School 3 meant that there was ample time for relationships between teacher and learner to develop before the ‘meatier’ aspects of RSE occurred:

We have the kids for 2 years which is great, because you’re developing that relationship even more, and I think that plays a role in delivering that and the questions that might come up because they do feel they’re in a trusted environment (Liz).

It is really quite interesting but as well good to be able to have that trust to have those conversations with the kids. And they trust what you say and its quite good. Makes for meaty conversations (Ana).

The importance of ‘knowing the learner’ is documented in local educational research such as Te Kotahitanga (Bishop et al., 2014) and Mana Ūkaipō (Highfield & Webber, 2021), and in health education contexts (Dixon, 2020). As Liz from school three mentions above, building trust between teacher and learner is an important pre-requisite for the sometimes sensitive topics covered in a subject such as RSE.

Considered as part of the teachers’ responses to the third workshop prompt (RSE for diverse ākonga) was the collection of student voice to inform planning and teaching (in RSE). The three schools participating in our research held different views, however, on the extent to which student voice was gathered and acted upon within their school. For School 1, student voice was the starting point for planning for teaching: “For us it’s just default, it’s like well it’s for them, so let’s just go to them first” (Rebecca). The participants from school three discussed the collection of student voice more broadly in the school setting in terms of upholding the mana of Māori learners:

I think we actually do quite a lot here to just, to ensure that the children that identify as Māori actually have that mana installed in them (Ana).

They’re acknowledged, recognised, celebrated (Liz).

We gather their voice (Alison).

To illustrate the complexity of seeking and acting upon student voice in the primary school, however, the participants from School 2 discussed the tensions surrounding collecting student voice to inform planning in RSE:

I would be nervous about collecting student voice too often because if we’re not prepared to use it—and there are many things we can’t use… I think it’s better off to choose what you’re asking really carefully or gather it a different way (Petra).

It’s like if you don’t survey them, you don’t know what they want, but if you know your students that you’re working with everyday then you shouldn’t have to survey them to know their needs (Christine).

And we’ve been talking about how a lot of our teaching here is responsive—not to the voice talking to us but listening and observing (Petra).

According to ERO (2018), “if schools are not regularly collecting information about what students want to learn… there is a risk that they are not meeting their students’ needs” (p. 17). Walker et al. (2021) also reinforce the importance of flexibility in teacher practice to meet students’ needs. It is therefore important for primary schools to find ways of seeking and integrating student voice that are workable for their contexts.

### Access to Wider Supports and Services

A final aspect of the village of people involved in RSE in primary schools is the array of wider supports and services that are accessed by schools. These are used to support leaders and teachers to develop their capability, as external providers that supported RSE in the classroom, and as providers of teaching and learning materials that teachers used for RSE. For the case study schools, supports that they accessed reflected the nature and character of their context. As noted by Wylie and MacDonald (2020), access to supports connected to student wellbeing such as social workers and health professionals are only funded for primary schools in communities underserved by social and economic systems. In our case study schools, only School 1 (a year 1–13 school) had access to counselling services on-site: “We have a school counsellor, and next year we’ll have two” (Rebecca).

Moving away from student support services towards supports to develop teacher capability, the participants from school 2 discussed how the Catholic advisors supported them to embed RSE in ways appropriate to the special character of their school:

The Catholic worldview sits alongside (the RSE guide), so everything’s taught through the Catholic worldview but acknowledges everything in that document… We’re quite happy for our Catholic RE advisors who have looked at it to bring in both together at the same time (Christine).

The on-going support and written guidance offered by the Catholic advisors was viewed as being invaluable to reassure the teachers and leaders at the school that their RSE practice aligned with the special character of the school.

In Schools 1 and 3, external supports were used to enhance teacher capability to teach RSE and to embed a safe and inclusive environment. Rebecca from School 1 spoke about her relationship with the regional schools co-ordinator from InsideOUT, who work to make Aotearoa a safer place for all rainbow young people:

We’ve had (co-ordinator) in this year. I just spoke with (person) and got the three new resources that aren’t actually officially out to the schools yet… I’m just having a flick through those at the moment and then (principal) is going to have a look.

This comment speaks to the fact that both Rebecca and her principal are interested in learning more about up-to-date effective practice in making the school safer for rainbow youth. This move is endorsed by messages in the RSE guide (Ministry of Education, 2020a) and reinforced by support from the Ministry of Education for the development of these resources for schools (InsideOUT, 2022). Further research, however, is needed into coverage of rainbow content in RSE, which to-date has been found to be lacking (Ellis & Bentham, 2021). It is important to acknowledge that this is an area of RSE that can be open to social, political, and cultural tensions, both in Aotearoa (Family First, 2021) and in other parts of the world (The Guardian, 2022). These tensions may have a role to play in teachers’ coverage and confidence in this aspect of RSE.

Another layer of support that can be accessed by schools are external providers who teach part or all of RSE in primary schools. Across the three schools, the participants discussed some use of external providers to support RSE teaching, but this was not extensive. Christine from School 2 explained how the public health nurse worked in partnership with the school to teach aspects of RSE:

She’ll do her role for that small bit of actual puberty or her wee brief that she’ll do, and then we do the rest of it. So, we’ve still got one section where the public health nurse will do and the rest of it we’re going to cover of all the ages and stages.

Christine went on to explain that the school uses external expertise to enhance learning rather than to replace the teacher in RSE learning: “I still think we’d possibly get an outside educator in here as well to really enhance it… because we’re not experts and we’ve got so much else to do”. This is a double-edged sword, however, as the final part of Christine’s point attests to—aspects of RSE learning may feel outside teachers’ areas of expertise, and time or opportunity for professional learning and development may not exist, thus requiring the need for external support on an on-going basis.

The question of who the best teacher is for RSE also arose. Christine from School 2 said that “if they’ve been with me from year 4, and they’ve grown up with their teacher—literally, they don’t want to talk about it with that person.” Whereas Alison from school three held an alternative view: “Whereas now it’s kind of done with the person that you trust—in your classroom environment.” The polarised views above resonate with research (Dixon, 2020; ERO, 2018) which argues that heterogenous views exist as to whether students are more comfortable being taught RSE by their classroom teacher or by an external provider. As noted by ERO (2018), this underlines the importance of capturing student voice in order to ascertain what ‘works’ for a given group of learners in a given context.

A final aspect in our case study schools relating to external supports and services connected to the use of teaching and learning resources for RSE, discussed as part of the second workshop extract (key learning in RSE). Family Planning’s (2018) *Navigating the Journey* resource was discussed by each school, although school two noted that they had to be careful with how they used their resources, given potential conflicts between the organisation and the Catholic worldview: “They have some amazing stuff, but it doesn’t fit with a Catholic school” (Christine). For Schools 1 and 3, however, *Navigating the Journey* (Family Planning, 2018) was the main resource used by all teachers for RSE. The resource is available in different volumes for year 1-year 10 learners, and each volume covers the same five themes. Hence, learning progression is evident across the years of the resource, as the following exchange from School 3 evidences:

I think one of the big parts of the Navigating the Journey that I’ve really appreciated is that you’re building a culture of inclusion and you’re basing it around the learner and you’re having conversations that are really important, from a variety of ages and its continued. I really like the fact that you build from a 5-year-old (Alison).

And it’s that progression (Ana).

It is interesting to note that *Navigating the Journey* (Family Planning, 2018) was the only teaching and learning resource that teachers knew about, despite the Ministry of Education producing two Curriculum in Action resources for RSE in 2017. This raises the question of how teachers and schools can stay up-to-date and informed about new resources to support teaching and learning, and how the Ministry of Education could be more proactive in communicating what they offer to schools.

## Conclusion

As evidenced by our analysis of findings from our collective case study, a wide range of people in the school community are involved in partnerships when it comes to RSE in the classroom and RSE-related issues in the wider school environment. While it can be challenging to invite whānau and student input into RSE, and to collaborate within and across schools, our findings indicate the power of voice, collaboration, and partnerships in helping to shape RSE practice, as advocated for by literature in the field (ERO, 2018; Johnson et al., 2014; Leahy et al., 2016; Ministry of Education, 2020a).

Collaboration with colleagues within or across schools, and support from those in governance and leadership positions in schools are important in order to provide conditions under which RSE can flourish, including the on-going development of teacher capability and confidence to plan and teach responsive learning experiences in RSE, which connects strongly to the guidance provided by the Ministry of Education (2020a) and the evaluative work of the ERO (ERO, 2018).

The requirement to consult with the community around the delivery of health education (including RSE) is unique to the subject, which presents both opportunities and challenges for schools. Adding a pandemic to the mix, implementing meaningful consultation opportunities may not always be easy. But as our findings demonstrate, teachers and schools are working to provide whānau a range of opportunities to have their say about the delivery of health education and RSE learning. Robinson et al. (2017) found that parents were overwhelmingly supportive of RSE, and this is reflected in our findings.

Below, we pose questions to spark thinking about the implications of our study for a range of members of the ‘village’:

- How can those in governance and school leadership work to support teachers, and work in partnership with whānau, students and wider agencies, to work towards a responsive RSE?
- How can school leaders and teachers collect and leverage off student voice in order to inform RSE practice?
- What partnerships could be (further) developed between colleagues across schools, and between teachers and wider support services and agencies, in ways that support effective practice in RSE?
- What possibilities for doing things differently in the future arise from the commentary expressed by our participants?

A methodological strength of our study was that the workshop part of the hui enabled opportunities for the participants to discuss with each other the strengths and the areas for development in their school, therefore providing data that aligned with the discovery and dream aspects of the Appreciative Inquiry cycle. Having pre-prepared prompts on specific aspects of practice that were connected to the RSE guide (Ministry of Education, 2020a) enabled rich conversations about strengths in existing practice, but also possibilities for the future. This conversation was more organic and participant-directed than the interviewer questions, thus adding a valuable layer to the hui. The workshop portions also yielded data that was comprised of exchanges between participants as they discussed the prompt points, rather than interviewer-interviewee responses that did not explore in as much detail the issue at hand.

A limitation of our small, exploratory study was that we were unable to implement the four stages of the 4D model for Appreciative Inquiry. Our future goal would be to work in partnership with teachers and senior leaders in order to not only discover and dream, but to design and reach a co-constructed destiny. A fully worked approach to Appreciative Inquiry, involves partnership and participation; people coming together to explore their world, which we were unable to achieve.

Our study has demonstrated that it indeed takes a village to plan for, and enact, a responsive RSE in primary schools that connects to known effective practice, and fulfils statutory requirements. We have shown that having conversations, and establishing and maintaining productive partnerships between the members of the village are not without their challenges, but are critical if RSE both inside and outside of the classroom is to live up to its potential and meet learners’ needs.

## Footnotes

1 We use this Māori term to refer to New Zealand.

2 We use whānau in this paper to refer to parents, caregivers, and family. The Māori term offers a broader understanding of ‘family’ and acknowledges the existence of a range of family structures in our world (Mutch & Collins, 2012).

3 The RSE guide uses the Māori term ‘ākonga to refer to learners. In terms of ‘diverse ākonga’ the RSE guide outlines guidance for ākonga Māori, Pacific ākonga, LQBTQI + ākonga and disabled ākonga. Our workshop prompt used excerpts for each of these groups of ākonga.
